# Visual Improvement Following Ozonetherapy in Dry Age Related Macular Degeneration; a Review

**Published:** 2013

**Authors:** Emma Borrelli, Velio Bocci

**Affiliations:** 1 Department of Biotechnologies, University of Siena, Italy; 2 Department of Physiology, University of Siena, Italy

**Keywords:** Ozone, Hydrogen peroxide, Alkenals, Antioxidant response element, Chronic Oxidative Stress

## Abstract

The dry form of ARMD is becoming a serious problem because of the rise in the number of old individuals. No effective therapy is available in dry ARMD except for the illusory oral administration of antioxidant vitamins. Despite scepticism in the medical community, the therapeutic effect of ozonetherapy had been evaluated since 1996. This evaluation has been based on specific biochemical, molecular and pharmacological reactions. Nevertheless a number of visual scientists continue to ignore ozonetherapy conservatively and prescribe only antioxidant vitamins. Two small clinical studies involving 217 patients have been performed at the University of Siena showing that ozonetherapy can stop the progression of the disease while improving the visual acuity and the well-being of the patient. Moreover, it seems that ozonetherapy is a safe procedure and tends to have an excellent compliance.

## INTRODUCTION

This is our fourth report [1-3] regarding the significant improvement of visual acuity in patients affected by the dry form of age related macular degeneration (ARMD). They have been treated twice weekly for several months with the autologous infusion of ozonated blood (O3-Autohemotherapy-O3-AHT).

During the last few years, this modality of treatment has been successfully performed in a number of patients in Italy and Germany. Although informed of the results, the most of ophthalmologists have remained controversies to perform the therapy. Only the dry form of ARMD can be treated by ozonetherapy and although it may not completely beneficial to increase visual acuity, this approach offers a buttress against the natural course of ARMD and improves quality of life [1,2].

ARMD might be attributed to aging, genetic predisposition, smoking, excessive exposure to sunlight, a blue iris, hyperopia vascular diseases with hypertension and possibly a nutritional lack of zinc and specific antioxidants such as lutein. It affects about 25% of people over 65 and is associated with a reduction of daily activities and independence. There are two forms of ARMD: a dry, atrophic form and a wet exudative form comprising neovascular formations. The dry form accounts for almost 90% of cases and the pattern of clinical features includes drusen, pigment clumping and/or retinal pigment epithelium (RPE) alterations and geographic atrophy. Patients develop a gradual visual loss over a few years with central visual scotomas slowly leading to complete loss of vision. The outer face of the retina is in contact with the Bruch’s membrane, which separates the vascular choroids from the RPE, which represents both the histological and functional connection with the photoreceptors situated in the outer layer. The RPE is vital to the integrity of the photoreceptors because it exerts crucial functions such as the daily phagocytosis of about 10% of the tips of the outer segments of the photoreceptors, the recycling of vitamin A and the transfer of oxygen and nutrients from the choroids to the photoreceptors and outer retina. The macula lutea and in its centre the foveola has the highest concentration of cones and is responsible for the visual acuity. For its metabolic requirement the foveola depends entirely on the choriocapillaris circulation because there are no retinal vessels and, among the various tissues of the body, it has the highest consumption of oxygen of all organs. The lack of oxygen rapidly leads to peripheral and/or central loss of vision by the degeneration of the neurosensorial cells [4]. Besides the critical aspects of ischemia, several deleterious processes such as bursts of free radicals, calcium ion induced damage and glutamate toxicity aggravate the progressive degeneration and loss of precious photoreceptors. Indeed there is a good correlation between C-Reactive protein serum level and ARMD implicating the role of a chronic inflammation and an intensive oxidative stress in the pathogenesis of the disease [5]. 

The wet form of ARMD is characterized by choroidal neovascular detachment of the RPE and fibrovascular disciform scarring associated with poor visual prognosis. The most recent and useful medical treatment is the intravitreal administration of monoclonal antibodies, particularly the ranibizumab (Lucentis) against the vascular endothelial growth factor. O3-AHT cannot be used in the wet form because it will accelerate the progression of the disease. The therapy is used to stop the natural course of the disease or at least to slow it down, but it cannot recover lost visual acuity and may have disturbing side effects [2].

In terms of epidemiological point of view, it must be kept in mind that in the UK there are about 250,000 partially sighted or blind patients and in the USA almost 10 million Americans are blind from ARMD [6].

## HYPOTHESIS

The anatomic-pathological situation can be dealt by the application of ozonetherapy. We have studied this problem for over two decades and we have clarified the biochemical, molecular and pharmacologic events [7-10]. It is interesting to know that ozone is ten-fold more soluble in the water of plasma than oxygen: this fact implies that mixing human blood with an equivalent volume of a gas mixture composed of about 96% O2 and 4% O3 leads to a very rapid solubilization of ozone, which, owing to its reactivity, reacts with hydrosoluble antioxidants (ascorbic acid, uric acid, trace of reduced glutathione) and with the unsaturated lipids carried out by albumin as follows:

R-CH = HC-R + O3 + H2O → H2O2 + 2 R-CHO

This means that in 2-3 minutes all the ozone has completely reacted and generated its messengers such as hydrogen peroxide and aldehydes derived from the unsaturated fatty acids peroxidation. The formation of aldehydes leads to the final formation of alkenals such as trans-4-hydroxy-2-nonenal (4-HNE from n-6) and a small quantity of trans-4-hydroxy-2-hexenal (from n-3) [11]. Consequently ozone acts simply as a pro-drug and generates these two messengers. The unionized hydrogen peroxide immediately enters into the mass of erythrocytes and activates glycolysis with ATP increase and, most important, enhances the production of 2,3 di-phospho-glygerate (2,3-DPG), which is able to shift to the right the oxyhemoglobin dissociation curve, thus increasing the release of oxygen in the ischemic areas such as the macula lutea and the foveola [12].

Although the alkenals are intrinsically toxic, owing to the minimal ozone concentration, are produced in the range of a few micromoles and undergo to either dilution, degradation by specific enzymes, elimination via the bile and urine but, most important, submicromolar quantities form adducts with either the Cys34 of albumin or the-SH group of reduced GSH [9]. This step represents for the patient, who has been infused with his or her own ozonated blood, a calculated and well tolerated oxidative stress because the albumin adducts transport and deliver the alkenals to cells of many organs [13-15]. Alkenals, inside the cell, binds to two-SH-groups (Cys272 and Cys288) of a large protein denominated Keap-1. Normally Keap-1 is bound to Nrf2 and, as many other transcription factors, is floating freely in the cell cytoplasm. This complex has a half-life of about 20 min because is continuously digested by the proteasome. However, when the two SH groups of the Keap1 have bound two molecules of 4-HNE, the protein Nrf2 is released and translocates into the cell nucleus where, after making an heterodimer with a small Maf protein, binds to the antioxidant response elements (ARE). This is the crucial event able to stimulate the upregulation of about 200 genes responsible for the transcription of a great number of antioxidant proteins (SOD, catalase, GSH-Px, GSH-Tr, etc) phase II enzymes and heme-oxygenase-1[16-18], which is a very protective enzyme. With the progress of ozonetherapy, these enzymes will be able to reverse the chronic oxidative stress induced by the chronic inflammation. At the same time, the enhanced oxygen release and possibly of lutein helps to stop the progress of ARMD. So far, millions of ozonated autohemotherapy, performed with small and precise ozone dosages, have never caused any side effects. In fact, most patients report a feeling of wellness which is likely due to a transitory increase of cortisol [17]. Lastly, ozonetherapy has also a minimal cost.

For the dry ARMD there are no useful therapies with the exception of the controversial oral administration of antioxidants such as vitamin A, C and E [5] with the addition of lutein and zeaxantin [19]. These compounds are certainly not harmful but at the best they may only delay the progression of the diseases after an average treatment period of at least six years, difficult to comply.

In terms of ozonetherapy procedure, depending upon the patient’s body weight, between 150-225 ml of blood are drawn by vacuum from an antecubital vein via a 19-21 G needle into a sterile glass, 500 ml bottle (Ozonosan, Iffezheim, Germany) containing 1 ml of 3.8% Na citrate solution as an anticoagulant for every 9 ml of withdrawn blood (9:1 ratio). A corresponding volume of a gas mixture (O2: 96%- O3: 4%), precisely measured regarding the ozone concentration via a spectrophotometrically reading on line at 253.7 nm (Hartley band), is immediately added with an initial ozone concentration of 20 mcg/ml gas per ml of blood. Thus for a total volume of 200 ml blood the total ozone dose is equivalent to 4.0 mg. Ozone is produced by a last generation ozone generator fed with only medical oxygen. The spectrophotometer must be checked by iodometric titration every year according the International ozone Association rule. The gas and blood are immediately and gently mixed, avoiding foam formation, for no longer than a few minutes and then the oxygenated-ozonated blood is infused into the donor patient in about 15 minutes. It is emphasized that the transfusion must be only autologous and is absolutely safe. The treatment is carried out twice weekly (either Monday and Thursday or Tuesday and Friday) for the initial 9-10 weeks. Normally the patient starts to note an improvement of visual acuity after 4-6 treatments. Afterwards it must be performed at least every 10 days for maintaining the therapeutic effects. It is important to note that the initial low ozone concentration is slowly upgraded up to no more than 50 mcg per ml of blood. The axiom: start low, go slow is proficiently used with this procedure. Indeed the oxidation properties of ozone represent a controlled oxidative stress for the patient and the low ozone concentrations are well tolerated because they yield a hormetic dose-response relationship [10]. The above maintenance therapy is absolutely necessary and it can last several years. The explanation is due to the fact that ozone therapy is based on precise biochemical basis and, if it is suspended, the therapeutic effects tend to fade and possibly disappear within 6-9 months. The patient must be informed that the biochemical reactions have a rather short memory but this is not a drawback because patients are very compliant once they have noted the improvement. Indeed the positive patient’s response is the best criterion to show that the therapy is effective.

## DISCUSSION

Two clinical trials have been performed by using ozonetherapy. The first was carried out by Bocci and Diadori [2] in the Department of ophthalmology of the University of Siena from 1996 to 2001 and included 77 patients. The trial was complicated because the clinical committee obliged us to evaluate a control group of patients treated with only oxygenated blood therapy. Although the request was theoretically correct, during a preliminary attempt only a minimal advantage had been noted. Today this request appears unethical because the oxygenation improvement is not due to the slow and transitory infusion of the hyperoxygenated blood because the simply oxygenated blood abundantly mixes with venous blood. The patient age ranged from 63 to 81 years old and each patient was treated for at least two years. In the treatment group an improvement in visual acuity more that 2 ETDRS lines was observed in 36 patients (66.6%), equal or less than 2 ETDRS lines in 18 patients (33.3%). In the control group an improvement in visual acuity more than 2 ETDRS lines was observed in 7 patients (30.4%), equal or less than 2 ETDRS lines in 16 patients (68.5%) These differences were statistically significant (chi-square). Laboratory tests regarding blood cells, coagulation tests, fibrinolysis tests, platelet tests, plasma proteins and plasma lipids tests either remained unvaried or improved.

The second trial performed by Borrelli et al. [3] enrolled 140 dry-ARMD patients from 2008 to 2011 with an age ranging from 59 to 82 years old. In this trial 70 patients underwent ozonetherapy while the control group (70 patients) assumed only the multivitaminic therapy. Ophthalmic examination was recorded for each eye at baseline and after 6 and 12 months for both groups. At 6 and 12 months, ozone treated eyes showed an average acuity change of -0.1 and of -0.2 logMar respectively, while control group eyes had a change of 0.2 and of 0.3, respectively: As far as the best corrected visual acuity (BCVA) changes over time, the data are: at 6 months none of the eyes treated with ozone had a deterioration in BCVA more than 2 lines: At the same interval, 16% of the control group eyes had more than 2 lines loss, 25% had more than 3 lines loss (P<0.05). As far as line improvements, at 6 months 4% of the treated group eyes had an improvement of 1 line compared to 0% of the control group patients. Again, at 12 months none of the treated group eyes showed a line loss more than 2 or 3 lines in BCVA compared with more than 2 line loss of 40% and more than 3 line loss of 38% in the control group patients (P<0.05). Moreover the plasma oxidative stress was significantly decreased after the treatment confirming the role of antioxidants enzymes in the increase and the reduction of the oxidative stress. Patients reported an improvement of their general conditions, particularly in terms of increased efficacy, mental concentration and memory as assessed by the National Eye Visual Function Questionnaire (NEI-VFQ) before and after the end of the study in all patients of group 1 and 2 [3].

## CONCLUSION

The fascinating aspect of ozonetherapy is its ability to trigger a number of defence mechanisms against ischemic and neurotoxic injury thus preventing the death of photoreceptors. These effects are summarized as follows: 

1) Improvement of blood rheology.

2) Improvement of the glycolytic pathways on erythrocytes. The increased concentration of ATP levels may facilitate a micro release at hypoxic sites.

3) Activation of the hexose-monophosphate shunt on erythrocytes with increased levels of 2,3-DPG particularly if the patient has had a low level. This change increases oxygen availability to hypoxic tissues due to a shift to the right of the HbO2 dissociation curve.

4) Vasodilation due to enhanced release of nitric oxide and prostacyclin.

5) Release of growth factors from platelets.

6) Of great importance is the upregulation of antioxidant enzymes, phase 2-proteins and heme-oxygenase-1 with release of CO and bilirubin.

The observation that visual acuity improves more rapidly in patients with an initial deficit than in almost blind patients strongly suggests that patients should be encouraged to start ozonetherapy as early as possible. It appears obvious that the therapy minimizes the death of photoreceptors. Finally there are other retinal degenerative disorders such as degenerative myopia, retinal vascular disorders due to diabetes, retinitis pigmentosa, recessive Stargardt’s disease, ischemic optic neuropathies and glaucoma. We would appreciate the international collaboration in this regard.

## DISCLOSURE

Conflicts of Interest: None declared.
